# 2200. The Antibiotic Steward's Nightmare: Long-Term Antibiotics in An Infectious Disease Practice

**DOI:** 10.1093/ofid/ofad500.1822

**Published:** 2023-11-27

**Authors:** Gabriel Karkenny, Megan Pao, Seleshi Demissie, Nikeith Shah, Bruce Hirsch

**Affiliations:** Long Island Jewish Medical Center, New Hyde Park, New York; Northwell, Manhasset, New York; Northwell, Manhasset, New York; Northwell, Manhasset, New York; Hoftsa Northwell School of Medicine, Manhasset, NY

## Abstract

**Background:**

The effects of long-term antibiotic use, including the frequency of recurrent infections and new-onset resistant infections, are not fully understood. The role of antibiotics in the disruption of the gut microbiota has been well-described, impacting insulin susceptibility and lipid deposition.

Prolonged antibiotic use may have potential to be implicated in obesity, diabetes, and hyperlipidemia. There may also be an effect on the incidence of secondary infections with new organisms, the frequency of breakthrough infections from the index organism, and the selection for resistant infections.

**Methods:**

This retrospective study evaluated 78 patients seen at an outpatient infectious disease practice. Inclusion criteria were patients with prescription of an oral antibiotic for 90 or more consecutive days between 9/1/18 and 12/31/2019, who utilize a health system pharmacy. Data was collected from patient charts at baseline prior to antibiotic initiation, and at time of most recent follow up after the initiation of antibiotic.

**Results:**

Cephalexin (37.2%), azithromycin (34.6%) and amoxicillin (12.8%) were the most common antibiotics prescribed. Mean patient age was 69.4 years. Indications for antibiotic use included NTM infections (34.6% of cases), prosthetic joint infections (21.8%), recurrent UTIs (10.3%), and prosthetic valve infection (7.7%). 23.4% of patients experienced breakthrough infection with the index organism, while 21.8% experienced new, unrelated infection during follow-up, of which 52.9% had a resistant organism isolated. Mean BMI at baseline was 26.9, and BMI at follow-up was 27.3. The difference between baseline and follow-up BMI was not statistically significant (p=0.177). A1C, LDL, and cholesterol rose from baseline to follow-up, but did not reach statistical significance.

**Conclusion:**

Patients on long-term antibiotics had a numerical increase in BMI, A1C, LDL, and cholesterol values following initiation, but those increases were not statistically significant. Recurrence of infection and emergence of resistance was common. The small sample size and limitation of studying patients from one practice within one health system pharmacy are limitations of the study. Further studies with larger sample sizes and comparative groups by diagnosis are needed.

**Disclosures:**

**All Authors**: No reported disclosures

